# Integrative analysis of genome-wide association studies of polyphenols in apple fruits identifies the MdDof2.4*-MdPAT10* module that promotes procyanidin accumulation

**DOI:** 10.1093/hr/uhae349

**Published:** 2024-12-12

**Authors:** Zhongxing Li, Cai Gao, Tianle Fan, Yilin Cui, Zeyuan Liu, Lei Li, Qian Qian, Mengjie Cheng, Xiangqiang Zhan, Chundong Niu, Fengwang Ma, Peizhi Yang, Qingmei Guan

**Affiliations:** State Key Laboratory for Crop Stress Resistance and High-Efficiency Production/Shaanxi Key Laboratory of Apple, College of Horticulture, Northwest A&F University, Yangling, Shaanxi 712100, China; College of Grassland Agriculture, Northwest A&F University, Yangling, Shaanxi 712100, China; College of Grassland Agriculture, Northwest A&F University, Yangling, Shaanxi 712100, China; State Key Laboratory for Crop Stress Resistance and High-Efficiency Production/Shaanxi Key Laboratory of Apple, College of Horticulture, Northwest A&F University, Yangling, Shaanxi 712100, China; College of Grassland Agriculture, Northwest A&F University, Yangling, Shaanxi 712100, China; State Key Laboratory for Crop Stress Resistance and High-Efficiency Production/Shaanxi Key Laboratory of Apple, College of Horticulture, Northwest A&F University, Yangling, Shaanxi 712100, China; State Key Laboratory for Crop Stress Resistance and High-Efficiency Production/Shaanxi Key Laboratory of Apple, College of Horticulture, Northwest A&F University, Yangling, Shaanxi 712100, China; State Key Laboratory for Crop Stress Resistance and High-Efficiency Production/Shaanxi Key Laboratory of Apple, College of Horticulture, Northwest A&F University, Yangling, Shaanxi 712100, China; State Key Laboratory for Crop Stress Resistance and High-Efficiency Production/Shaanxi Key Laboratory of Apple, College of Horticulture, Northwest A&F University, Yangling, Shaanxi 712100, China; State Key Laboratory for Crop Stress Resistance and High-Efficiency Production/Shaanxi Key Laboratory of Apple, College of Horticulture, Northwest A&F University, Yangling, Shaanxi 712100, China; State Key Laboratory for Crop Stress Resistance and High-Efficiency Production/Shaanxi Key Laboratory of Apple, College of Horticulture, Northwest A&F University, Yangling, Shaanxi 712100, China; State Key Laboratory for Crop Stress Resistance and High-Efficiency Production/Shaanxi Key Laboratory of Apple, College of Horticulture, Northwest A&F University, Yangling, Shaanxi 712100, China; College of Grassland Agriculture, Northwest A&F University, Yangling, Shaanxi 712100, China; State Key Laboratory for Crop Stress Resistance and High-Efficiency Production/Shaanxi Key Laboratory of Apple, College of Horticulture, Northwest A&F University, Yangling, Shaanxi 712100, China

## Abstract

Polyphenols represent a significant class of nutrients in apples, contributing to human health and well-being. Among these, procyanidins stand out as the most prevalent polyphenolic compounds in apple fruits. These compounds are abundant in wild apples and generally low in modern apple cultivars. Therefore, it is crucial to identify and recover genetically lost genes that regulate polyphenol accumulation in order to improve the apple quality. To achieve this, we conducted a genome-wide association study (GWAS) on 15 traits related to polyphenol content, utilizing 134 *Malus* accessions. We identified 1204 marker–trait associations (MTAs) and 840 candidate genes, including known polyphenol biosynthetic and regulatory genes, such as *MYB7*, *TT4*, and *HCT/HQT*. Notably, we pinpointed a protein *S*-acyl transferase 10 (PAT10), which is significantly associated with procyanidin content. Through experiments with transgenic calli, we determined that apple (*Malus domestica*) MdPAT10 positively regulated procyanidin accumulation. Furthermore, we identified a 51-bp insertion variant (In-868) on the promoter of the *PAT10*, which influences its expression. Both a yeast one-hybrid (Y1H) assay and an electrophoretic mobility shift assay (EMSA) revealed that MdDof2.4 was able to bind to the promoter of *MdPAT10* containing In-868 (*MdPAT10_pro_^In-868^*), but not to the promoter of *MdPAT10* without In-868 (*MdPAT10_pro_*). Moreover, MdDof2.4 promoted *MdPAT10* (with *MdPAT10_pro_^In-868^*) expression and increased procyanidin accumulation in fruits. Overall, our results enhance the understanding of the biosynthetic regulation of apple polyphenols and provide a theoretical foundation and genetic resources for breeding apple varieties with optimal polyphenol content.

## Introduction

Apple (*Malus domestica*), a member of the Rosaceae family, is one of the most widely cultivated and consumed fruit crops worldwide. In 2022, global production of apples exceeded 95 million tonnes [1]. Apples are particularly recognized as a rich dietary source of antioxidants due to their high levels of polyphenols and other bioactive compounds that contribute to human health and well-being. Polyphenolic compounds, a class of secondary metabolites with multiple phenolic hydroxyl groups, exhibit significant antioxidant and free radical scavenging activities [2, 3]. However, while polyphenolic compounds are abundant in wild apples, their levels are generally lower in modern apple cultivars [4, 5].

The biosynthetic pathway of polyphenolic compounds is highly complex and involves intricate molecular mechanisms controlled by key enzymes and diverse transcription factors [6, 7]. For example, key enzymes like anthocyanidin synthase (ANS), anthocyanidin reductase (ANR), and leucoanthocyanidin reductase (LAR), are pivotal rate-limiting steps in polyphenol biosynthesis [8, 9]. The MYB–bHLH–WD40 (MBW) complex is a well-established transcriptional regulator that plays a crucial role in anthocyanin biosynthesis [10], as well as other families of transcription factors, such as WIP, NAC, and WRKY. Notably, the overexpression (OE) of *MdWRKY11* can increase the accumulation of flavonoids in apple callus tissues [11]. Sun *et al*. [12] reported that NAC52 binds to the promoters of *MYB9* and *MYB11*, directly regulating LAR to promote anthocyanin and procyanidin (PC) biosynthesis in apple. Furthermore, research indicates that the levels of PC are affected by post-translational modifications, such as histone methylation [13], ubiquitination [14], and SUMOylation [15].

Palmitoylation is a dynamic and reversible post-translational modification that regulates the localization, activity, stability, and interactions of proteins. It plays a key role in cellular signal transduction, metabolism, apoptosis, and other biological processes [16]. TIP1 (Tip Growth Defective 1), a member of the palmitoyltransferase family identified in plants, regulates protein hydrophobicity in *Arabidopsis*, affecting protein–membrane binding, signal transduction, and intracellular vesicle transport [17]. Previous studies have shown that AtPAT10, exhibiting *S*-acyl transferase activity, enhanced the reproductive capacity of *Arabidopsis* by regulating the cell enlargement and division [18, 19]. AtPAT24 can complement the phenotype of the yeast PAT-deficient mutant (*akr1*), thus restoring the morphological and temperature-sensitive defects of the *akr1* mutant [20]. Li *et al*. [21] found that AtPAT14 is influenced by salicylic acid signaling with its mutants exhibiting a reduced biomass phenotype and negatively regulating leaf senescence. Furthermore, members of the C subfamily of palmitoyltransferases (PAT19, PAT20, and PAT22) interact with the key cytoplasmic receptor kinase BSK1 and catalyze its palmitoylation modification of BSK1 in *Arabidopsis* [22]. In apples, MdPAT16 can palmitoylate MdCBL1, thus stabilizing its protein level and regulating sugar accumulation and salt tolerance [23]. To date, the involvement of palmitoylation in the regulation of PC biosynthesis is largely unknown.

In this study, we performed genome-wide association study (GWAS) analyses of 15 traits of polyphenol content in 134 *Malus* accessions to determine the genetic basis of polyphenol accumulation and identified marker–trait associations and candidate genes. Additionally, we conducted a comprehensive analysis of the association between the identified protein *S*-acyltransferase 10 (MdPAT10) and PC accumulation. These findings enhance our understanding of the regulatory pathway of apple polyphenol biosynthesis and provide genetic resources for further breeding of apple varieties with optimal polyphenol content.

## Results

### Polyphenolic compound content in *Malus* accessions

**Figure 1:**
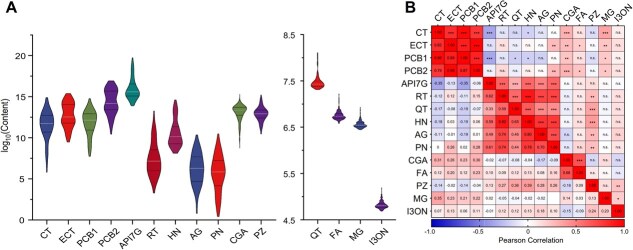
(A) Distribution and (B) correlation of the content of 15 polyphenolic compounds from 134 *Malus* accessions. The left diagonal section of the rectangle illustrates the Pearson correlation between the polyphenolic compounds, while the right diagonal section depicts the significance of these correlations. Statistical analyses were performed using the Student’s *t*-test, with significant differences are indicated by ^*^P < 0.05, ^**^P < 0.01, or ^***^P < 0.001. N.s. indicates no significant difference. CT, catechin; ECT, epicatechin; PCB1, procyanidin B1; PCB2, procyanidin B2; API7G, apigenin 7-glucoside; RT, rutin; QT, quercetin; HN, hyperin; AG, astragalin; PN, prunin; CGA, chlorogenic acid; FA, ferulic acid; PZ, phloridzin; MG, methyl gallate; I3ON, isorhamnetin 3-O neohesperidoside.

We collected fruit flesh from 134 *Malus* accessions, comprising 23 wild accessions (Wild), 32 *Malus sieversii* accessions (*M. sieversii*), 52 cultivars (Cultivar), and 27 hybrids (Hybrid), and determined the content of 15 polyphenolic compounds by liquid chromatography–mass spectrometry (LC–MS) (Fig. 1a, Fig. ,S1, and Table S1). The content of these 15 polyphenols in apple flesh showed significant variation. Among them, apigenin 7-glucoside (API7G), procyanidin B2 (PCB2), and epicatechin (ECT) had the highest average contents of 19.5, 4.6, and 0.9 mg/g, respectively. In contrast, isorhamnetin 3-O neohesperidoside (I3ON) and methyl gallate (MG) had the lowest content (Fig. 1a and Table S2). Rutin (RT), quercetin (QT), prunin (PN), and 12 other polyphenolic compounds exhibited coefficients of variation (CV) >60%, with RT having the highest CV of 383%, followed by QT and PN with CVs of 269% and 248%, respectively (Table S2).

Correlations were observed among the content of the 15 polyphenolic compounds. The content of catechins (CTs), ECTs, procyanidin B1 (PCB1), and PCB2 showed significant correlations (Fig. 1b). Additionally, significant correlations were found between the content of API7G, RT, QT, HN, astragalin (AG), and PN (Fig. 1b), which is consistent with the network of biosynthetic metabolism pathways of these compounds.

We observed significant differences in the content of the 15 polyphenolic compounds among the three groups (Wild, *M. sieversii*, and Cultivar; Fig. S2). Higher content of polyphenols was found in *M. sieversii* and Wild groups, while lower content of polyphenols was observed in the Cultivar group. Notably, the content of PCB2 and API7G showed significant difference in *M. sieversii* and Wild groups compared with the Cultivar group (Fig. S2), which indicates that the phenolic content is altered during the domestication and improvement of apples.

### Population structure and linkage disequilibrium

We performed single-nucleotide polymorphism (SNP) analysis on whole-genome resequencing data from 134 *Malus* accessions, utilizing the Genome Analysis ToolKit (GATK) Best Practices Workflow. Through this analysis, we identified a total of 10 987 271 high-confidence SNPs, corresponding to 17.58 SNPs per kilobase (kb), for subsequent analyses of genetic variation and population structure (Table S1 and Table S3).

**Figure 2:**
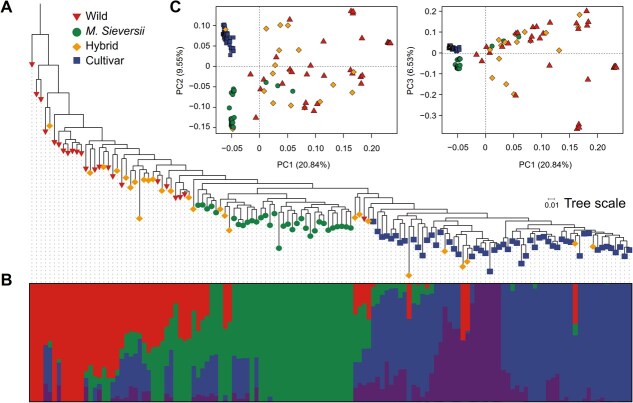
Genomic relationship and population structure of 134 *Malus* accessions. (A) Clades and groups, including Wild, *M. sieversii*, Hybrid, and Cultivar, as illustrated by a phylogenetic tree. (B) Population structure of model-based clustering when *K* = 4. (C) PCA of Wild, *M. sieversii*, Hybrid, and Cultivar. Different color markers represent the groups as in (A).

To further investigate the genetic structure of the 134 *Malus* accessions, we performed population structure analyses. Firstly, we constructed a phylogenetic tree based on the neighbor-joining (NJ) method with 1000 bootstraps. The phylogenetic topologies indicate that the Wild, *M. sieversii*, and Cultivar are three monophyletic taxa, while the hybrids are primarily located in the central region between Wild and *M. sieversii* (Fig. 2a). Among them, the Cultivar group was genetically closer to the *M. sieversii* than to the Wild group. Subsequently, we performed admixture analyses. When *K* = 4 (best *K*, Fig. S3), Wild, *M. sieversi,i*, and Cultivar groups were clearly segregated from each other. *Malus sieversii* has a relatively independent genetic background and contributed genetically to the formation of cultivars to a certain extent (Fig. 2b). This finding is consistent with the previously described origins of domestication and dispersal routes of apples [24, 25]. Ultimately, we examined these accessions by SNP-based principal component analysis (PCA). We similarly observed that the Cultivar is clearly separated from the Wild and *M. sieversii*, closer to *M. sieversii* (Fig. 2c). In addition, PCA demonstrates that hybrids were mainly distributed in the middle of the Wild and *M. sieversii*, with some hybrids found between *M. sieversii* and Cultivar, or located within one of these groups (Fig. 2c). These results are in consistency with those of phylogenetic relationships (Fig. 2a). The patterns of the population structure suggest that the genetic origins of Hybrid indeed derive from genetic mixing between different species of the *Malus* genus.

Rapid linkage disequilibrium (LD) decay was observed in the Wild group, while the LD decay process in *M. sieversii*, Cultivar, and Hybrid groups was relatively slow (Fig. S4a).

In addition, we found differences in nucleotide diversity (π) and population fixation index (*F_ST_*) among the four groups. The highest π was found in the Hybrid group, followed by the Wild group, whereas the lowest π was found in the *M. sieversii* and Cultivar groups (Fig. S4b). The *F_ST_* values showed that the cultivars were genetically distant from the *M. sieversii* and Wild groups, which aligns with the findings from the phylogenetic tree and PCA (Fig. 2a and 2c). These results imply a degree of population differentiation among the Wild, *M. sieversii*, and Cultivar groups, which might contribute to the differences in phenolic content among *Malus* accessions.

### Genome-wide association study on 15 polyphenols

**Figure 3:**
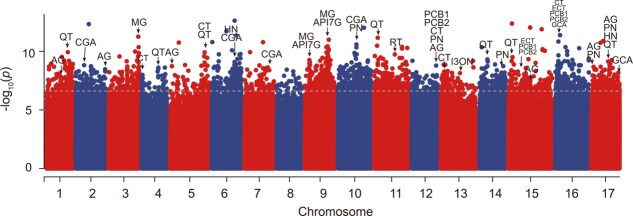
Manhattan plot of GWAS for 15 traits in *Malus* accessions. CT, catechin; ECT, epicatechin; PCB1, procyanidin B1; PCB2, procyanidin B2; API7G, apigenin 7-glucoside; RT, rutin; QT, quercetin; HN, hyperin; AG, astragalin; PN, prunin; CGA, chlorogenic acid; FA, ferulic acid; PZ, phloridzin; MG, methyl gallate; I3ON, isorhamnetin 3-O neohesperidoside.

Based on the dataset of 10 987 271 high-confidence SNPs from 134 resequenced *Malus* accessions, we conducted GWAS analyses of 15 traits of individual polyphenol content. Acknowledging the significant impact of population genetic structure on GWAS efficacy [26], we corrected the effect of population stratification on the GWAS using PCA and kinship matrix. Subsequently, we employed the mixed linear model (MLM) method, as implemented in the GEMMA software [27], and identified 1204 marker–trait associations (MTAs) and 840 candidate genes (Fig. 3, Table 1, Fig,. S5, and Table S4).

**Table 1:** Lead SNPs and related known genes significantly associated with 15 polyphenolic traits according to the GWAS for the 134 *Malus* accessions

**Trait**	**Chromosome**	**Lead SNP**	**-log**_**10**_ **(*P*)**	**PVE (%)**	**Major allele**	**Minor allele**	**Gene symbol**
CT	Chr04	2 589 849	7.07	30.62	G	A	*WD40*
	Chr16	38 580 219	7.13	30.70	C	T	*bHLH*
ECT	Chr15	19 343 501	6.93	15.22	C	G	*DHHC*
	Chr16	2 107 668	6.96	30.10	T	C	*MYB7*
	Chr16	3 334 171	6.82	32.40	T	A	*AP2*
	Chr16	3 455 183	6.95	33.10	G	C	*TCP12*
PCB1	Chr16	2 107 668	6.82	29.10	T	C	*MYB7*
	Chr16	3 404 850	6.57	22.51	T	C	*LAR*
PCB2	Chr09	5 720 628	7.25	34.17	A	C	*WD40*
API7G	Chr09	27 442 887	7.79	22.30	C	G	*WRKY40*
	Chr09	28 410 789	6.78	18.88	G	A	*HCT*
RT	Chr03	15 265 020	5.3	15.53	T	C	*WIP3*
	Chr07	26 738 882	5.27	16.06	C	T	*TT5*
QT	Chr04	19 713 978	8.28	11.92	A	G	*TT4*
	Chr05	18 136 875	8.84	13.42	C	T	*UGT73B3*
	Chr14	21 450 469	8.39	12.13	A	G	*Dof*
	Chr15	4 360 622	12.38	15.57	G	A	*MYB84*
HN	Chr01	11 202 493	6.28	18.66	C	T	*MYB93*
AG	Chr07	614 305	7.53	23.02	G	T	*bHLH*
	Chr15	17 358 160	7.13	21.62	C	A	*TT2*
	Chr15	21 422 536	8.58	27.55	T	G	*bZIP44*
	Chr17	11 145 873	6.77	20.63	A	T	*UGT85A3*
PN	Chr10	26 219 946	7.61	32.89	A	T	*ABI3*
	Chr14	17 647 147	6.9	29.47	T	C	*MYB103*
	Chr17	3 777 291	8.55	26.11	T	C	*MYB94*
CGA	Chr16	29 456 547	7.17	31.19	G	A	*WD40*
	Chr17	28 263 534	7.94	36.38	A	G	*bZIP61*
	Chr17	27 376 190	6.21	26.87	G	T	*HCT/HQT*
	Chr17	27 726 498	5.94	24.81	C	T	*4CL2*
FA	Chr16	34 045 701	7.27	17.71	C	T	*MYB36*
PZ	Chr05	19 940 664	6.87	30.23	G	A	*WD40*
MG	Chr09	27 197 923	7.75	16.12	A	T	*CCR1*
	Chr09	27 454 208	9.46	27.66	T	C	*WRKY40*
	Chr09	28 479 365	7.91	16.02	G	A	*HCT/HQT*
I3ON	Chr16	20 082 085	6.22	15.49	T	A	*C2H2*

PVE, phenotypic variation explained; CT, catechin; ECT, epicatechin; PCB1, procyanidin B1; PCB2, procyanidin B2; API7G, apigenin 7-glucoside; RT, rutin; QT, quercetin; HN, hyperin; AG, astragalin; PN, prunin; CGA, chlorogenic acid; FA, ferulic acid; PZ, phloridzin; MG, methyl gallate; I3ON, isorhamnetin 3-O neohesperidoside.

**Figure 4:**
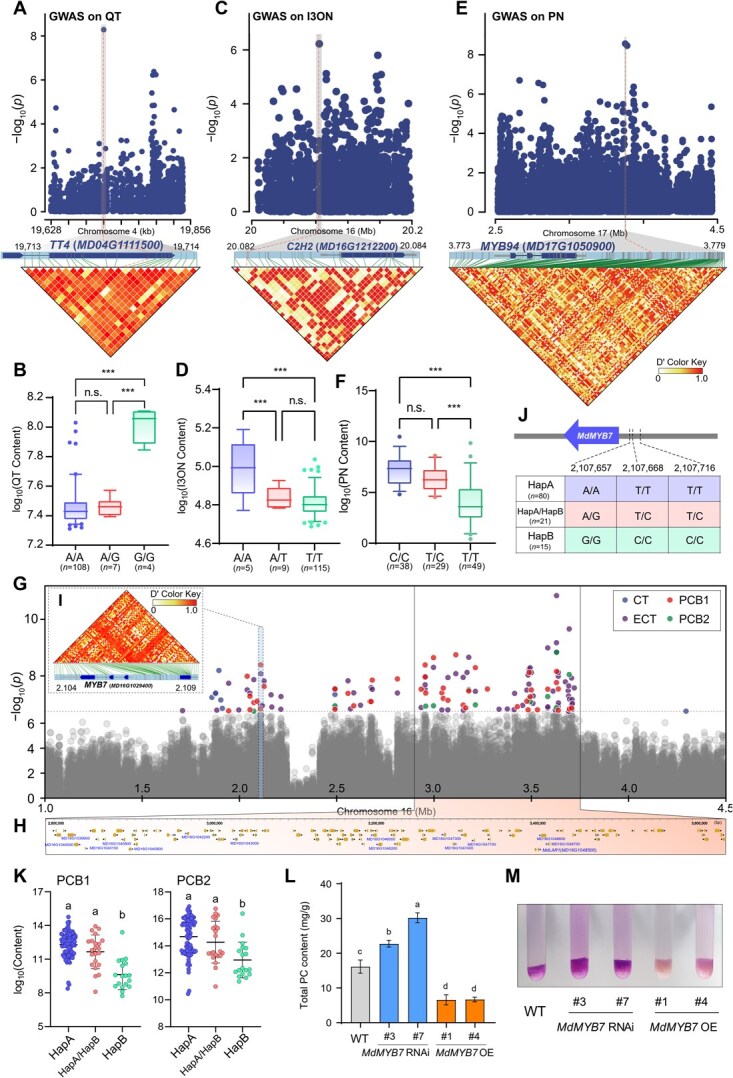
GWAS-based dissection of polyphenol traits and causative genes. (A–F) Integrated analysis of GWAS signals, LD blocks, and polyphenolic content for quercetin (QT), isorhamnetin 3-O neohesperidoside (I3ON), and prunin (PN) were conducted. Local Manhattan plot (top) and LD heat map (bottom) illustrate the GWAS signal for QT (A), I3ON (C), and PN (E) traits. Boxplots for QT (B), I3ON (D), and PN (F) content are presented based on the lead SNPs. The dash line indicates the lead SNPs at Chr4:19713978 (A), Chr16:20082085 (C), and Chr17:27376190 (E). Statistical analyses were performed using the Student’s *t*-test, with statistically significant differences indicated by ^***^P < 0.001 in (B, D, and F). N.s. indicates no significant difference. (G) Local Manhattan plot of catechin (CT), epicatechin (ECT), procyanidin B1 (PCB1), and procyanidin B2 (PCB2) on Chromosome 16. (H) Genes located in polyphenol hotspot QTL, where text-labeled genes are known loci in the phenolic synthesis pathway. (I) LD heat map surrounding the peak signal for ECT and PCB1 traits. (J) Haplotype analysis based on GWAS significant sites (Chr16:2107657, Chr16:2107668, and Chr16:2107716) on the *MdMYB7* promoter. (K) Boxplots for PCB1 and PCB2 content are presented according to the haplotypes in (J). (L) Content of total procyanidin (PC) in WT, *MdMYB7* overexpression (OE), and RNA interference (RNAi) lines of apple calli. Error bars indicate SD (*n* = 15). One-way analysis of variance (ANOVA , Duncan’s test) was performed, with statistically significant differences indicated by lowercase letters in (K and F). (M) DMACA staining of apple calli (WT, *MdMYB7* OE, and RNAi lines).

We identified known key loci involved in polyphenol regulation at these significant MTAs and candidate genes. A notable signal on chromosome 4, linked to QT content, was located within the *MD04G1111500* gene (Fig. 4a). This gene encodes chalcone synthase (CHS), a key enzyme in the biosynthesis of QT [28]. The lead SNP (Chr4:19713978, *P* = 5.20 × 10^−9^, PVE = 11.92%) was associated with QT content and strongly linked with *MD04G1111500* (Fig. 4a)*.* Furthermore, *Malus* accessions with the homozygous G/G allele had higher content of QT than those with the homozygous A/A allele or heterozygous A/G allele (Fig. 4b).

*MD16G1212200*, located near the GWAS signal of I3ON on chromosome 16, encodes a C2H2 zinc finger protein (Fig. 4c). The lead SNP (Chr16:20082085, *P* = 6.00 × 10^−7^, PVE = 15.49%) was located upstream of *MD16G1212200* and linked with the gene body. Accessions with the homozygous A/A allele had higher content of I3ON than those with the homozygous T/T allele or heterozygous A/T allele (Fig. 4d). Moreover, a signal for PN on chromosome 17 is located downstream of *MD17G1050900* (Fig. 4e), which encodes an R2R3-type MYB transcription factor. Accessions with the homozygous T/T allele had lower content of PN than those with the homozygous C/C allele or heterozygous T/C allele (Fig. 4f). Additionally, a nonsynonymous SNP (Chr17:27376190, *P* = 6.17 × 10^−7^, PVE = 26.87%) was strongly associated with chlorogenic acid (CGA) content and is located within the *hydroxycinnamoyl-CoA shikimate/quinate hydroxycinnamoyl transferase* (*HCT/HQT*) gene (Table 1), which is the key rate-limiting enzyme for CGA biosynthesis [29].

PCs are a class of polyphenolic compounds consisting of CT or ECT units bound in various amounts. In this study, we identified genetic markers associated with the content of CT, ECT, PCB1, and PCB2 with 30, 88, 54, and 20 significantly associated SNPs, respectively (Table 1 and Table S4). Specifically, within the interval of 1.0–4.5 Mb on chromosome 16, we identified a GWAS hotspot region strongly correlated with these four traits (CT, ECT, PCB1, and PCB2 content; Fig. 4g and 4h). This hotspot region coincided with a previously reported hotspot area [30, 31].

Remarkably, three SNPs (Chr16:2107657, Chr16:2107668, and Chr16:2107716) located in the promoter region of the *MdMYB7* were significantly associated with PC content (Fig. 4i, 4j, and Table S4). *MYB7* encodes an R2R3-type MYB transcription factor, and its homologous protein in *Arabidopsis* (AtMYB7) acts as a repressor of flavonol biosynthesis [32]. Moreover, haplotype analysis revealed three principal haplotype combinations: HapA as ATT (*n* = 80), HapB as GCC (*n* = 15), and HapA/HapB (heterozygous) as ATT/GCC (*n* = 21) (Fig. 4j). Accessions carrying HapA or HapA/HapB exhibited significantly higher content of CT, ECT, PCB1, and PCB2 than those carrying HapB (Fig. 4k and Fig. S6).

We investigated the role of *MdMYB7* in apple PC biosynthesis by generating transgenic OE and RNA interference (RNAi) apple calli of *MdMYB7* (Fig. S7). By measuring the total PC content, we found that the *MdMYB7* OE lines had significantly lower PC than wild type (WT). Conversely, the MdMYB7 RNAi lines demonstrated significantly elevated PC levels relative to the WT (Fig. 4l). Furthermore, dimethylaminocinnamaldehyde (DMACA) staining confirmed these findings (Fig. 4m). These findings indicate that *MdMYB7* acts as a negative regulator of PC accumulation in apples.

### PAT10 plays a critical role in PC biosynthesis

**Figure 5:**
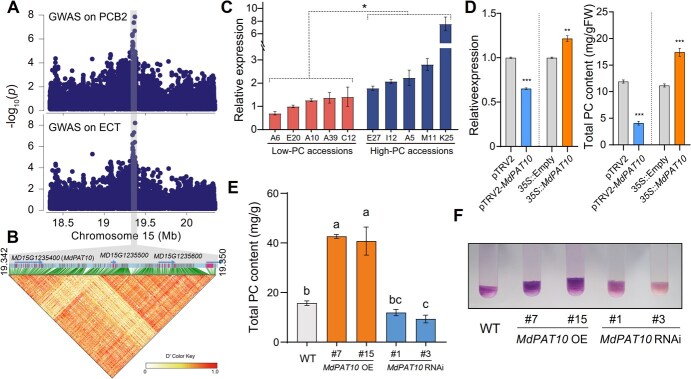
The *MdPAT10* enhances procyanidin accumulation in apple. (A) Local Manhattan plot surrounding the GWAS signal for procyanidin B2 (PCB2, top) and epicatechin (ECT, bottom). (B) LD heat map surrounding the peak signal for PCB2 and ECT traits. (C) qRT-PCR analysis of *MdPAT10* in low- and high-PC content apples. (D) PC content in the transiently overexpressed or silenced *MdPAT10* calli. *35S:MdPAT10* or pTRV2-*MdPAT10* were transiently transformed in ‘Qin Guan’ fruit at 100 DAF. Gene expression of *MdPAT10* was measured by qRT-PCR analysis (left). Statistical analyses were performed using the Student’s *t*-test, with statistically significant differences indicated by ^*^P < 0.05, ^**^P < 0.01, or ^***^P < 0.001 in (C and D). N.s. denotes no significant difference. Error bars indicate SD (*n* = 3 for qRT-PCR assay, and *n* = 20 for PC content assay). (E) PC content determination of WT and transgenic calli of *MdPAT10* OE and RNAi. Error bars indicate SD (*n* = 15). Statistical analyses were performed using the one-way ANOVA, with statistically significant differences indicated by different lowercase letters. (F) DMACA staining of apple calli (WT, *MdPAT10* OE, and RNAi lines).

Through GWAS of PCB2 and ECT, we pinpointed a significant peak associated with the *PAT10* gene on chromosome 15 (Fig. 5a and 5b). Signal peaks also appeared near the *PAT10* in the GWAS of CT and PCB1, but these peaks were not statistically significant (Fig. S8). Sequence homology analysis revealed that *PAT10* encodes the protein *S*-acyl transferase, whose function is critical for plant metabolism [18, 19]. Furthermore, through the phylogenetic tree and protein sequence alignment, we established that PAT10s are highly conserved in flowering plants (Fig. S9), suggesting that *PAT10* might be an ancient gene with fundamental roles in flowering plants. Importantly, we found that the expression level of *PAT10* was significantly higher in high-PC accessions than low-PC accessions (Fig. 5c).

Subsequently, we determined the function of *PAT10* in apple PC biosynthesis. Initially, we found that the transiently overexpressed *MdPAT10* in apple fruits resulted in a significant increase in the expression of *MdAPT10* as well as in the total PC content, in comparison to control fruits (Fig. 5d). Additionally, virus-induced gene silencing (VIGS) of *MdPAT10* in apple fruits showed a decrease in the expression of *MdPAT10*, along with a corresponding decrease in the total PC content compared to the control (Fig. 5d). Moreover, we generated *MdPAT10* OE and RNAi lines of apple calli (Fig. S10). The total PC content was significantly higher in the *MdPAT10* OE calli than that in the WT, while it was notably reduced in the *MdPAT10* RNAi calli relative to the WT (Fig. 5e). Meanwhile, DMACA staining further verified the above results (Fig. 5f). Collectively, these findings indicate that *MdPAT10* plays a positive regulatory role in the biosynthesis of PCs in apples.

### Natural variation in the *PAT10* promoter is associated with PC content in apples

**Figure 6:**
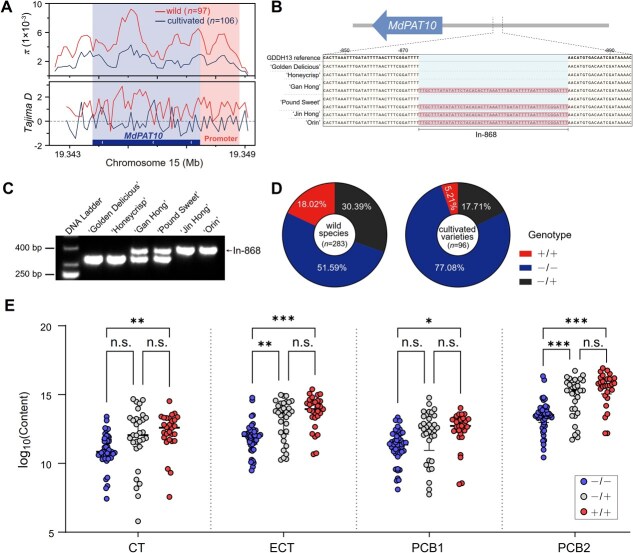
In-868 on *MdPAT10* promoter is significantly associated with PC content. (A) Nucleotide diversity (*π*) and Tajima’s *D* analyses of *MdPAT10* and its promoter region within the wild and cultivated apples. (B) Sanger sequencing and (C) PCR analysis of the *MdPAT10* promoter region in apple varieties. (D) Genotype distributions of In-868 in wild species and cultivated varieties. Source data in Fig. S11. (E) The content of CT, ECT, PCB1, and PCB2 of 134 *Malus* accessions with or without In-868. Statistical analyses were performed using the Student’s *t*-test, with statistically significant differences indicated by ^*^P < 0.05, ^**^P < 0.01, or ^***^P < 0.001. N.s. indicates no significant difference. In (D and E), +/+ represents accessions carrying homozygous In-868; −/− indicates accessions without homozygous In-868; −/+ denotes accessions carrying heterozygous In-868.

Population genetic analyses revealed a footprint of domestication selection on the *PAT10* gene. Nucleotide diversity (*π*) and Tajima’s *D* were calculated by using 97 wild and 106 cultivated samples that we previously documented [24]. Within the *PAT10* gene and its promoter region, *π* was higher in wild samples than in cultivated samples (Fig. 6a), indicating a reduction in genetic diversity among cultivated apples. Moreover, in the promoter of the *PAT10* gene, Tajima’s *D* value was greater than zero in wild samples, while in cultivated samples, it was less than zero. This disparity suggests the presence of selection pressure during the domestication process (Fig. 6a).

Subsequent analysis of the *PAT10* promoter by Sanger sequencing and polymerase chain reaction (PCR) analysis identified a 51-bp insertion variant, designated In-868, located at −868 bp upstream of the *MdPAT10* start codon. This variant was present in the ‘Jin Hong’ and ‘Orin’ varieties, but not in the ‘Golden Delicious’ and ‘Honeycrisp’ varieties (Fig. 6b and 6c). Furthermore, In-868 showed a heterozygous pattern in ‘Gan Hong’ and ‘Pound Sweet’ varieties (Fig. 6b and 6c). PCR analysis using additional 283 wild species and 96 cultivated varieties revealed notable differences in the distribution pattern of In-868 between wild species and cultivated varieties (Fig. 6d and Fig. S11). Specifically, the proportion of In-868 in wild species was 18.02%, whereas in cultivated varieties, it was only 5.21% (Fig. 6d and Fig. S11). This observation suggests that the In-868 genotype may have undergone negative selection during the domestication and improvement of apple varieties.

Remarkably, we found that accessions with In-868 had significantly higher levels of CT, ECT, PCB1, and PCB2 than those without In-868 (Fig. 6e). These results suggest that the In-868 variant is associated with PC accumulation in apples.

### Interaction of MdDof2.4 with *MdPAT10_pro_^In-868^* increases *MdPAT10* expression and PC accumulation

**Figure 7:**
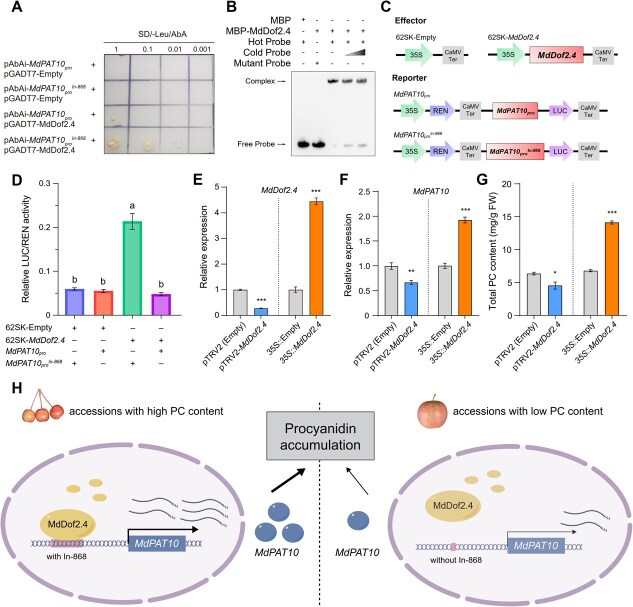
Interaction of MdDof2.4 with *MdPAT10_pro_^In-868^* increases the expression of *MdPAT10* and the accumulation of PC. (A) The Y1H assay shows the binding of MdDof2.4 to the insertion-containing promoter of *MdPAT10* (*MdPAT10_pro_^In-868^*). (B) EMSA demonstrates the binding of MdDof2.4 to the 5′-AAAAG-3′ motif in *MdPATA10_pro_^In-868^*. The hot probe is a biotin-labeled fragment containing the 5′-AAAAG-3′ motif; while the cold probe is a nonlabeled competitive probe, prepared at concentrations 150- and 250-fold, respectively, of the hot probe; the mutant motif is 5’-CCCCC-3′. (C) Diagrams of the effector and reporter constructs. (D) Interaction between MdDof2.4 and *MdPAT10_pro_^In-868^*, as determined by dual luciferase reporter assays in *Nicotiana benthamiana* leaves. Data are presented as the mean ± SE (*n* = 9). Statistical analyses were performed using the one-way ANOVA, with statistically significant differences indicated by different lowercase letters. (E–F) qRT-PCR analysis of *MdDof2*.4 (E) and *MdPAT10* (F) genes in WT, transiently overexpressed, or silenced *MdDof2.4* fruits. *35S: MdDof2.4* or pTRV2-*MdDof2.4* were transiently transformed in ‘Gan Hong’ fruit at 100 DAF. (G) Determination of the PC content in WT, transiently overexpressed, or silenced *MdDof2.4* fruits. Error bars indicate SD (*n* = 20). Statistical analyses were performed using the Student’s *t*-test, with statistically significant differences indicated by ^*^P < 0.05, ^**^P < 0.01, or ^***^P < 0.001 in (E–G). N.s. denotes no significant difference. (H) A proposed working model of the regulation of PC accumulation by the MdDof2.4-*MdPAT10* module in apples.

Our analysis of the In-868 natural variant sequence using the PlantRegMap website revealed that it contains a typical binding site (5′-AAAAG-3′) for MdDof2.4 (Table S5). A yeast one-hybrid (Y1H) assay confirmed that MdDof2.4 had the ability to bind to the In-868 on the *MdPAT10* promoter (*MdPAT10_pro_^In-868^*), while it did not bind to the *MdPAT10* promoter without In-868 (*MdPAT10_pro_*) (Fig. 7a). Additionally, an electrophoretic mobility shift assay (EMSA) demonstrated that the recombinant MdDof2.4 protein binds to the *MdPAT10_pro_^In-868^* fragment containing 5′-AAAAG-3′ (hot probe with biotin labeling). The binding of MdDof2.4 to the hot probe was competed off by unlabeled cold probe (150- and 250-fold) (Fig. 7b), indicating the specificity of the MdDof2.4-*MdPAT10_pro_^In-868^* interaction. Moreover, a dual-luciferase reporter gene assay (Dual-LUC) showed that the combination of MdDof2.4 and *MdPAT10pro^In-868^* could activate the expression of *LUC* (Fig. 7c and 7d), suggesting a strong binding affinity between MdDof2.4 and *MdPAT10pro^In-868^*. These results demonstrate that MdDof2.4 binds specifically to *MdPAT10_pro_^In-868^*, but not to *MdPAT10_pro_*.

The transient OE of *MdDof2.4* in the fruits of the cultivar apple ‘Gan Hong’ (harboring the In-868, Fig. 6b and 6c) promoted the expression of *MdPAT10* (Fig. 7e and 7f) and increased the total PC content (Fig. 7g). Additionally, silencing *MdDof2*.4 in ‘Gan Hong’ apple fruits by the VIGS technique resulted in a significantly reduced expression of *MdPAT10* and a significant reduction of the total PC content, compared to the fruits transformed with the empty vector (Fig. 7e–g). These findings suggest that MdDof2.4 is capable of promoting *MdPAT10* expression and thereby enhancing PC accumulation (Fig. 7h).

## Discussion

Polyphenolic metabolites in apples exhibit variation influenced by factors such as varieties, tissue types, growth periods, and environmental conditions [5, 33, 34]. Nevertheless, genetic variability remains the primary determinant of their content in plants [35, 36]. Previous studies have indicated that during the domestication and improvement of apples, there has been an increase in fruit size and sugar content, while the polyphenol content has decreased [4, 37]. In the study, we analyzed the content of 15 phenolic compounds across 134 *Malus* accessions and found that phenolic content was higher in wild accessions and relatively lower in domesticated apples, which is consistent with the trend observed during apple domestication and improvement [25, 37]. Through GWAS technology, we investigated the factors governing polyphenol biosynthesis, aiming to provide genetic resources for breeding apple varieties with enhanced health-promoting properties.

Association analyses of polyphenol content traits with genetic markers have revealed key regions involved in the regulation of polyphenol synthesis. For example, the 1.0- to 4.5-Mb interval on *Malus domestica* chromosome 16 has been identified as a hotspot quantitative trait locus (QTL) for polyphenol biosynthesis, containing several key genes closely related to this pathway, including *LAR1* and *MYB7* [30, 31]. Additionally, MBW complex has regulatory functions in the flavonoid biosynthetic pathway [10, 38]. For instance, in the ‘Yanfu 8’ apple variety, *MdMYB114* and *MdbHLH3/33* are involved in the formation of the MBW complex, promoting anthocyanin biosynthesis [39]. Furthermore, it has been demonstrated that in a variety of plants, including tomato, coffee, potato, and artichoke, HCT/HQT is a rate-limiting regulatory enzyme in the CGA biosynthetic pathway [40–42]. This finding was also confirmed in our study, where GWAS analyses based on CGA content similarly identified the HCT/HQT motif as a key site.

PCs represent a significant portion of the polyphenolic compounds found in apples and are predominantly synthesized through the flavonoid pathway. Studies have revealed that the flavonoid content can vary by 10 to 100 times among different apple varieties [35]. Although the biosynthetic pathway of PCs in apple has been largely elucidated, there remains a relative scarcity of studies focusing on their molecular regulatory mechanisms, particularly regarding post-translational modifications. Notably, palmitoylation, an important post-translational modification of proteins, plays a role in various localization and signaling pathways. Jiang *et al*. [23] demonstrated that MdPAT16 regulates sugar metabolism and salt tolerance in apple. However, the role of PATs in regulating the synthesis of phenolic compounds, such as PCs, has yet to be reported.

In this study, we found that the module of MdDo2.4-*MdPAT10* regulates the PC accumulation. As a transcription factor, MdDof2.4 activates the expression of *MdPAT10*, which in turn increases the levels of PCs and their precursors in apple. Specifically, the In-868 variant in the *MdPAT10* promoter region enhances the binding affinity of MdDof2.4, thereby promoting the expression of *MdPAT10*. Conversely, the absence of the In-868 results in the loss of the binding site for MdDof2.4, leading to decreased expression of *MdPAT10*. However, the specific molecular mechanisms remain to be investigated, particularly the effect of MdPAT10-mediated palmitoylation on PC biosynthesis.

## Conclusion

In summary, our study revealed the distributional characteristics of polyphenol content within the *Malus* genus and identified key enzymes and transcription factors involved in regulating polyphenol accumulation through GWAS. Additionally, we confirmed the significant role of MdDof2.4-*MdPAT10* module in the regulation of PC biosynthesis. These findings enhance our understanding of the regulatory network of apple PC biosynthesis and provide gene resources for breeding of new apple varieties.

## Materials and methods

### Plant material collection

In this study, we collected the fruits of 134 *Malus* accessions from the Apple Germplasm Resource Garden at the Horticultural Farm of the Northwest A&F University (NWAFU, Yangling, China). The accessions included 23 wild (Wild), 32 *M. sieversii* (*M. sieversii*), 52 cultivars (Cultivar), and 27 hybrids (Hybrid) (Table S1). These accessions had been subjected to whole-genome resequencing based on the Illumina platform in a previous study [24], and the average sequencing depth of the samples was ~18.62-fold coverage (Table S1).

For measuring polyphenolic metabolites, three to five fruits with uniform ripeness from at least two trees for one accession were collected as one biological replicate. Three biological replicates were used for each accession. In addition, young leaves of 283 wild species and 96 cultivated varieties, used for identifying In-868 through PCR, were gathered from the Apple Germplasm Resource Garden at the Horticultural Farm of the Northwest A&F University (NWAFU, Yangling, China).

### Extraction and measurement of polyphenolic metabolites

Polyphenols were extracted as previously described by Zhang *et al*. [43]. Briefly as follows: first, a 0.05 g sample of apple powder was taken and placed in a 1.5 ml polypropylene (PP) tube. Then, 1 ml of extraction solution was added, which consisted of a mixture of methanol, deionized water (ddH2O), and formic acid in a ratio of 25:24:1. Next, the tubes were sonicated in an ultrasonic cleaner for 20 min, and then the tubes were placed on an oscillator for 20 min. After shaking was completed, the tubes were placed in a centrifuge and centrifuged at 10 000 g for 15 min to separate the solid residue from the extraction solution. Subsequently, the supernatant was aspirated using a syringe and filtered through a 0.22-μm nylon filter (Osmonics, Fisher Scientific, Pittsburgh, USA). The filtered extraction was collected and suitably diluted 5-folds and then stored in amber glass vials for LC–MS analysis.

The HPLC (AB SCIEX ExionLC™ AC System) combined with a triple-quadruple linear ion trap mass spectrometer (Triple Quad™ 5500) was used for the precise analysis of polyphenol compounds. The chromatographic conditions were as follows: (i) Chromatographic column: InertSustain AQ-C18 column (4.6 mm × 150 mm, 5 μm, GL Sciences, Tokyo, Japan), with the column temperature set at 30°C. (ii) Flow rate: 300 μl/min. (iii) Solvent system: the mobile phase solutions were water with 0.1% formic acid (A) and 100% methanol (B). (iv) Gradient elution process: 0–1 min, 75% A and 25% B; 1–5 min, linear gradient to 5% A and 95% B; 5–6.5 min, maintain 5% A and 95% B; 6.5–12 min, return to initial conditions, 75% A and 25% B. (v) Injections for samples and standards: 5 μl.

The main parameters of the mass spectrometry were as follows: (i) Ion source: electrospray ionization (ESI). (ii) Ion source temperature (TEM): 600°C. (iii) Ion-spray voltages (IS): 5500 V. (iv) Nebulizer gas (GS1): 60 psi. (v) Heater gas (GS2): 60 psi. (vi) Curtain gas (CUR): 35 psi. (vii) Scanning mode: multireaction monitoring (MRM) was adopted as the method for efficient fragmentation and selective detection of polyphenol metabolites.

### Population structure analysis

Raw reads were trimmed using FASTP software (version 0.20.0) [44] and then mapped to the *M. domestica* ‘Golden Delicious’ reference genome (GDDH13 v1.1) by using BWA-MEM (version 0.7.17, https://github.com/lh3/bwa) with default parameters. Then, duplicated reads were detected and removed with the Picard tools (version 2.1.1) (http://broadinstitute.github.io/picard). SNP calling was performed using GATK (version 3.8) Best Practices workflow [45] as previously described [46].

An NJ tree was constructed using VCF2Dis software (version 1.47, https://github.com/BGI-shenzhen/VCF2Dis) on the basis of a distance matrix with 1000 bootstrap replicates. The web-based tool iTOL (https://itol.embl.de) was used to visualize the phylogenetic tree. PCA was performed using smartpca program embedded in EIGENSOFT (version 6.1.4) [47], and the first three eigenvectors were plotted. Population structure was assessed utilizing ADMIXTURE (version 1.3) [48] which is a model-based clustering method for inferring population structure. Genetic cluster (*K*) values ranging from 2 to 12 were predefined, and 20 replicates were executed for each *K* to estimate standard errors.

### Linkage disequilibrium analysis

In this study, PopLDdecay software (version 3.41) [49] was used to calculate the LD decay of SNPs in four groups (Wild, *M. sieversii*, Cultivar, and Hybrid) and all sample set (All). The parameter settings used were as follows: MaxDist = 20 Kb, MAF = 0.05, Het = 0.8.

### Genome-wide association study

In the study, we constructed a panel of association maps containing 134 *Malus* accessions. After genotype imputation, we identified 10 987 271 SNPs with a minimum allele frequency (MAF) >5%, which composed the genotype dataset for GWAS. In addition, we performed log transformation to the phenotypic data for the 15 traits to ensure that the phenotypes conformed to a normal distribution for use as phenotypes in GWAS.

We used the MLM of GEMMA software (version 0.98.5) [27] to explore the association between genotype and phenotype. To correct for the potential effects of population structure and kinship on the association analyses, we considered the population structure and kinship of the samples as cofactors in the model. The kinship correlation matrix among samples was calculated based on SNPs using a built-in program (−gk 2 parameter) in the GEMMA software. Besides, we used the first three principal components (PC1, PC2, and PC3) of PCA as covariates to further control for group stratification.

The Genetic Type I error calculator (GEC) software (version 0.2, https://pmglab.top/gec) was used to perform an accurate count of effective markers in the SNP dataset, which ultimately confirmed a total of 5 501 094 effective markers (*n_e_*). Then, we set a threshold of 1/*n_e_* to identify statistically significant association loci based on the false discovery rate (FDR) criterion [50]. We identified neighboring genes located within 5 kb upstream and downstream of the leading SNPs as significantly associated genes and obtained functional annotations of these genes through homology alignment to further identify candidate genes.

### Haplotype block analysis

We analyzed haplotype blocks surrounding significantly trait-associated SNPs and candidate genes (*TT4*, *C2H2*, *MYB94*, and *MdMYB7*) using the LDBlockShow software (version 1.40) [51] and genotyped them. The association of haplotype genotypes with trait phenotypes was evaluated using one-way analysis of variance (ANOVA) statistics followed by Duncan’s multiple comparison test.

### Vector construction and genetic transformation

To generate overexpressing *MdPAT10* or *MdMYB7* apple calli, the coding sequences (CDS) of *MdPAT10* and *MdMYB7* were cloned and introduced into the pK2GW7 vector, respectively. To obtain RNA-interfering *MdPAT10* or *MdMYB7* apple calli, fragments (200 bp) of *MdPAT10* or *MdMYB7*, respectively, were cloned and introduced into the pK7GWIWG2D vector. The resulting recombinant vectors were transformed into *Agrobacterium tumefaciens* strain EHA105, respectively. Then, *A. tumefaciens* carrying the plasmid was transformed into apple calli (‘Orin’), which were used as the WT for genetic transformation.

### Transient expression assay

To obtain transiently transformed apple fruits to overexpress the *MdPAT10* and *MdDof2.4* genes, we transformed recombinant plasmids pK2GW7-MdPAT10 and pK2GW7-MdDof2.4 into *A. tumefaciens* strain GV3101. The transformed strain was allowed to stand under light-avoidance conditions for 2–4 h and was subsequently injected into fruits of ‘Qin Guan’at 100 days after flowering (DAF). Quantitative real-time PCR (qRT-PCR) was used to detect the expression levels of *MdPAT10* and *MdDof2.4* after 7 days.

To generate *MdPAT10* and *MdDof2.4* gene-silenced transient transformation Apple materials, a VIGS assay was performed according to the previously described method [52]. Briefly, 200-bp fragments of *MdPAT10* and *MdDof2.4* were introduced into the pTRV2 vector, respectively. The pTRV1 plasmid, empty vector pTRV2, pTRV2-MdPAT10, and pTRV2-MdDof2.4 were transformed into *A. tumefaciens* strain GV3101, respectively. The four aforementioned strains (pTRV1, pTRV2-MdPAT10, pTRV2-MdDof2.4, and pTRV2) were cultured to OD_600_ = 0.5, and then pTRV1 was mixed with three other (pTRV2-MdPAT10, pTRV2-MdDof2.4, and pTRV2 empty vectors) strains, respectively, in equal volume. After standing for 2–4 h without light, the mixture was injected into ‘Gan Hong’ and ‘Qin Guan’ apple fruits, respectively, at 100 DAF. qRT-PCR was performed to analyze the expression levels of *MdPAT10* and *MdDof2.4* after 7 days.

### Staining and determination of PC

Procyanidin extraction and assay were performed according to the instructions in the Plant Procyanidins Content Assay Kit from Solarbio (product no. BC1350). Staining of apple calli was referred to previously reported [53], and the apple calli was stained using DMACA reagent [1% (w/v) ethanol: 6 M HCl (1:1, v/v)] for 2 h and then observed and photographed for documentation.

### qRT-PCR

RNA was extracted from apple calli or fruits using RNAprep Pure Plant PlusKit (DP441, TIANGEN, Beijing, China) in accordance with the manufacturer’s instructions. For qRT-PCR analysis, 1 μg of total RNA was reverse transcribed to first-strand cDNA with oligo-dT using the HiScript III RT SuperMix for qPCR kit (R323–01, Vazyme, Nanjing, China). ChamQ Universal SYBR qPCR Master Mix (C601, Vazyme, Nanjing, China) was used to perform qRT-PCR on a LightCycler® 480 II Real-Time System (Roche, Mannheim, Germany). *Malate dehydrogenase* (*MdMDH*) was used as a reference gene to calculate relative expression by the 2^-ΔΔCt^ method, with three biological and three technical replicates for each condition. All primers used are listed in Table S6.

### Selective sweep

We calculated the nucleotide diversity (π) [54] and Tajima’s *D* values [55] for the gene body, upstream, and downstream regions of the *MdPAT10*. SNP variation data from Chen *et al*. [24] for wild (*n* = 97) and cultivars (*n* = 106) was used, and the calculations were carried out using the VCFtools (version 0.1.17) [56] in the sliding-window approach, with the window size set to 1000 bp and a step size of 100 bp. R package (ggplot2) was used for the visualization of the calculated data.

### Y1H assay

Sequences of two promoters (*MdPAT10_pro_* and *MdPAT10_pro_^In-868^*) were submitted to the Plant Transcriptional Regulatory Map (PlantRegMap) database [57] for analysis of transcription factor binding sites. The results showed a potential binding site for MdDof2.4 on In-868 of the promoter.

To validate the interactions of MdDof2.4 with *MdPAT10_pro_* or *MdPAT10_pro_^In-868^*, we used a Y1H. Briefly, sequences without and with In-868 of *MdPAT10* promoter were constructed into the pAbAi vector and transformed into the Y1H yeast strain, which was inhibited from self-activation in SD/-Ura/AbA medium (AbA was used to inhibit self-activation). Subsequently, Y1H yeast strains containing pAbAi-*MdPAT10_pro_* and pAbAi-*MdPAT10_pro_^In-868^* were made into receptor cells, and the pGADT7-MdDof2.4 plasmid was transferred into the receptor cells, and pGADT7-Empty was transferred as a negative control. The transformed yeast cells were screened on SD/−Leu/AbA medium.

### Dual-luciferase reporter assay

The CDS sequence of *MdDof2.4* was cloned from a cDNA library of ‘Golden Delicious’ apple leaves and constructed into the pGreenII62-SK vector. Meanwhile, two promoter variants of the *MdPAT10* (*MdPAT10_pro_* and *MdPAT10_pro_^In-868^*) were also constructed into the pGreenII0800-LUC vector, respectively. Subsequently, these recombinant vectors were transferred into *A. tumefaciens* strain GV3101 containing pSoup, respectively. Suspensions of different combinations of pGreenII62-SK and pGreenII0800-LUC recombinant vector strains were mixed at a ratio of 1:1 and injected into 4-week-old tobacco leaves. Leaves were collected after 3 days of coinfiltration, and firefly luciferase (LUC) and renilla luciferase (REN) signals were quantified using the Dual Luciferase Reporter Gene Assay Kit (11402ES60, Yeasen, Shanghai, China).

### Electrophoretic mobility shift assay

The DNA-binding domain sequence of *MdDof2*.4 was cloned into pMAL-c5X (MBP) expression vector and subsequently transformed into *Transetta* (DE3) Chemically Competent Cell (CD801–02, TransGen, Beijing, China). Subsequently, positive monoclones were selected and amplified in culture, and the recombinant proteins were induced to be expressed by 0.5 mM IPTG. The cells were crushed using an ultrasonic instrument, followed by recombinant protein purification using Amylose resin. Additionally, the probes used for the EMSA experiments were synthesized by Sangon Biotech (Shanghai, China). EMSA experiments were performed on the purified proteins according to the guidelines of the Lightshift Chemiluminescent EMSA kit (20 148, Thermo Scientific, Pittsburgh, USA).

### Statistical analysis

Differences between two samples were analyzed with the use of a two-tailed Student’s *t*-test. For multiple comparisons, differences were analyzed using one-way ANOVA followed by Duncan’s multiple comparison test. Statistical analyses were performed using IBM SPSS Statistics 24.

## Supplementary Material

Web_Material_uhae349

## Data Availability

The raw whole-genome resequencing data of 134 *Malus* accessions were retrieved from the NCBI Sequence Read Archive (SRA) with the accession number PRJNA728537, and details in Table S1. Sequence data used in this article can be found in GenBank under the following accession numbers: XM_008339732.3 (MdPAT10); PQ100214 (MdDof2.4); XM_008354691.3 (MdMYB7).
