# Supplementary material for: Integrative analysis of genome-wide association studies of polyphenols in apple fruits identifies the MdDof2.4-MdPAT10 module that promotes procyanidin accumulation
Source: Hortic Res. 2024 Dec 12;12(3):uhae349. doi: 10.1093/hr/uhae349 (PMC11890028; doi:10.1093/hr/uhae349)
Supplement: Web_Material_uhae349 [file web_material_uhae349.zip › 12.SupplementalFigues.Revised.20241201.pdf]

Supplemental Figures for

Integrative Analysis of Genome-wide Association Studies of Polyphenols in Apple Fruits Identifies the MdDof2.4-*MdPAT10* Module That Promotes Procyanidin Accumulation

Fig. S1. Scatterplot matrix of 15 individual polyphenols of 134 *Malus* accessions.

Fig. S2. Content of polyphenols in three groups (Wild, *M. sieversii*, and Cultivar) in this study.

Fig. S3. Plot of ADMIXTURE cross-validation (CV) error for *K* values from 2 to 12.

Fig. S4. Population diversity and genetic differentiation analysis.

Fig. S5. Manhattan plots and quantile-quantile (Q-Q) plots for 15 individual polyphenol content traits.

Fig. S6. Boxplots for catechin (CT) and epicatechin (ECT) content followed the haplotypes in Fig. 4J.

Fig. S7. Relative expression of *MdMYB7* in *MdMYB7* RNAi (A) and *MdMYB7* OE (B) calli by qRT-PCR.

Fig. S8. Local Manhattan plot surrounding the GWAS signal for procyanidin B1 (PCB1, top) and catechin (CT, bottom).

Fig. S9. PAT10 protein sequence analysis in several plant species.

Fig. S10. Relative expression of *MdPAT10* in *MdPAT10* OE (A) and *MdPAT10* RNAi (B) calli by qRT-PCR.

Fig. S11. Detection of the In-868 variation in the *MdPAT10* promoter in wild species and cultivated varieties.

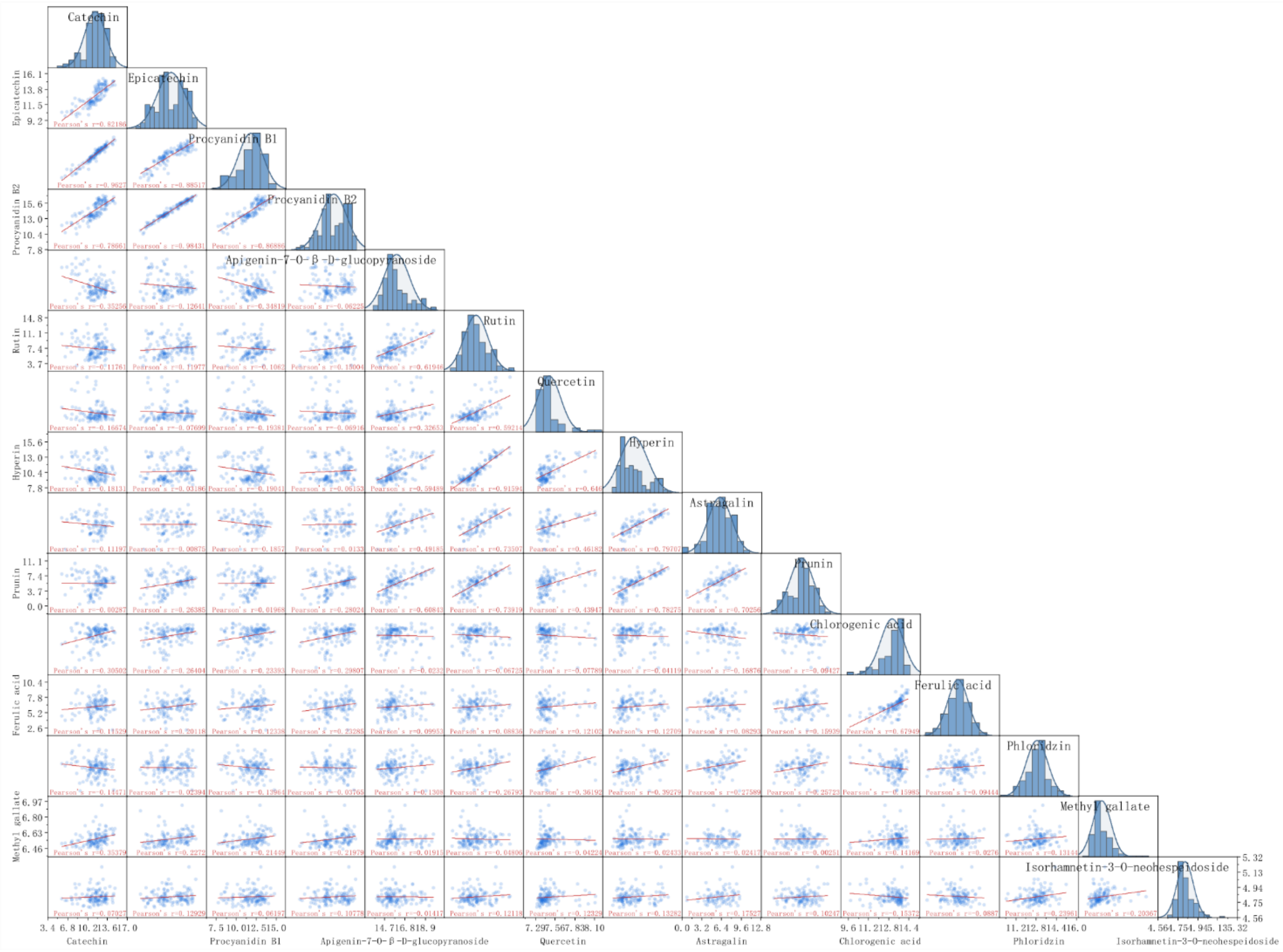

Fig. S1. Scatterplot matrix of 15 individual polyphenols of 134 *Malus* accessions.

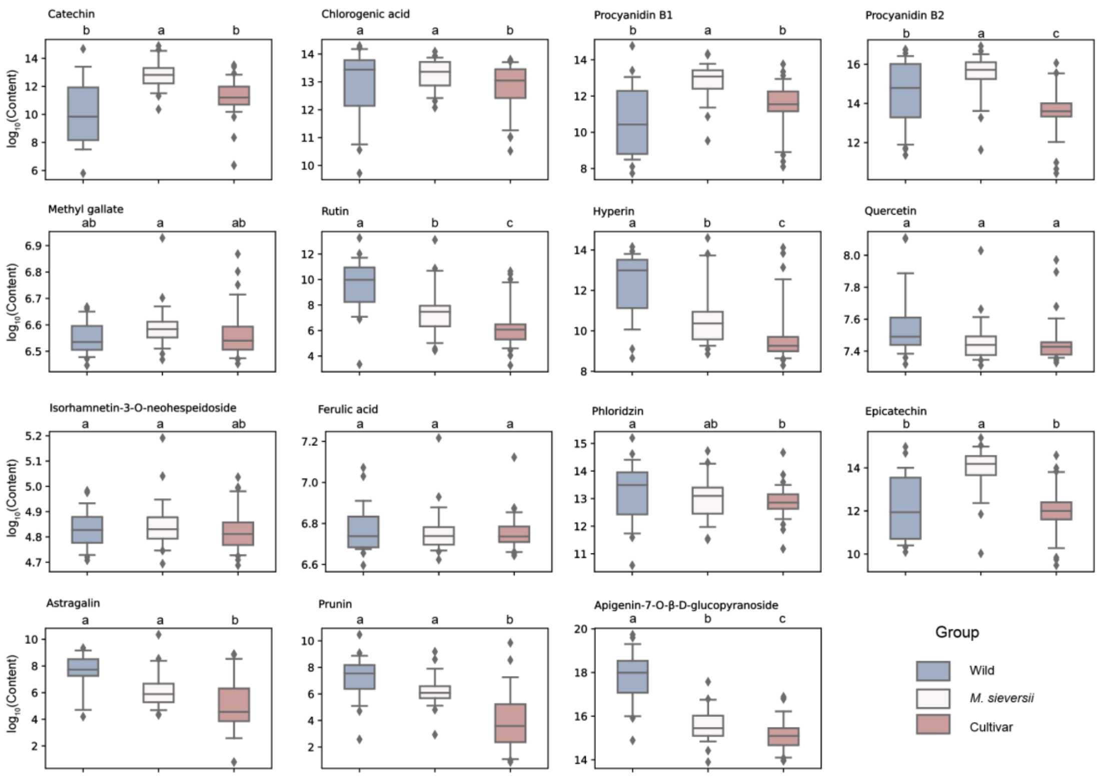

Fig. S2. Content of polyphenols in three groups (Wild, *M. sieversii*, and Cultivar) in this study.

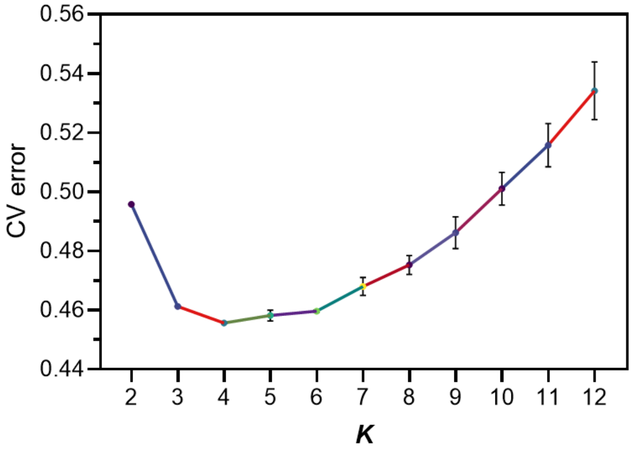

Fig. S3. Plot of ADMIXTURE cross-validation (CV) error for *K* values from 2 to 12. When *K*=4, the CV error value is minimised.

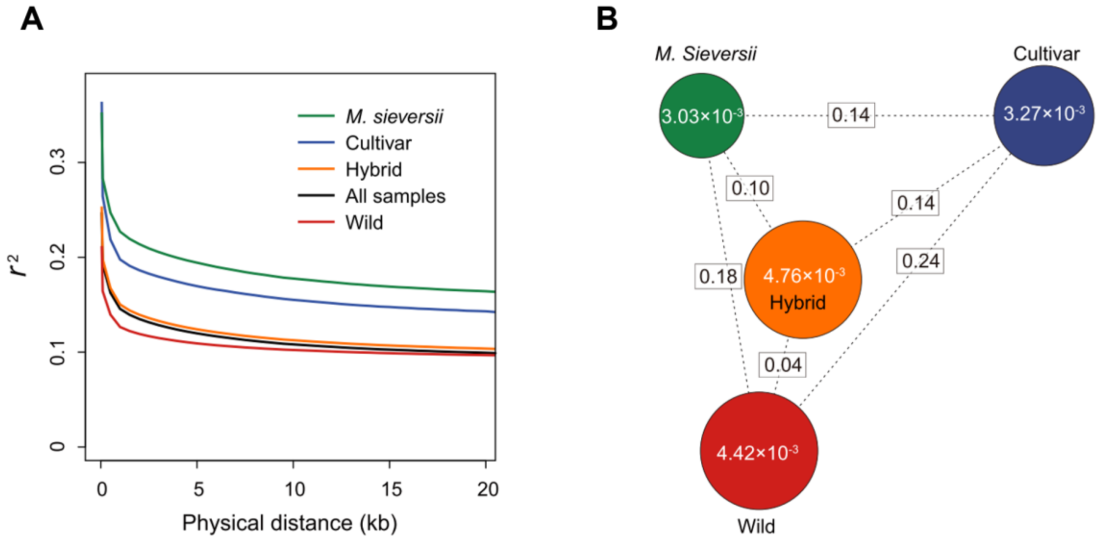

Fig. S4. Population diversity and genetic differentiation analysis. (**A**) Linkage disequilibrium (LD) decay of Wild, *M. sieversii*, Hybrid, and Cultivar, and All samples, measured by the coefficient of determination (r^2). (**B**) Summary of nucleotide diversity (π) and fixation index (F_{ST}) of Wild, *M. sieversii*, Hybrid, and Cultivar groups. The value in each circle represents the nucleotide diversity for the group, and the value on each line indicates the population differentiation among each two groups.

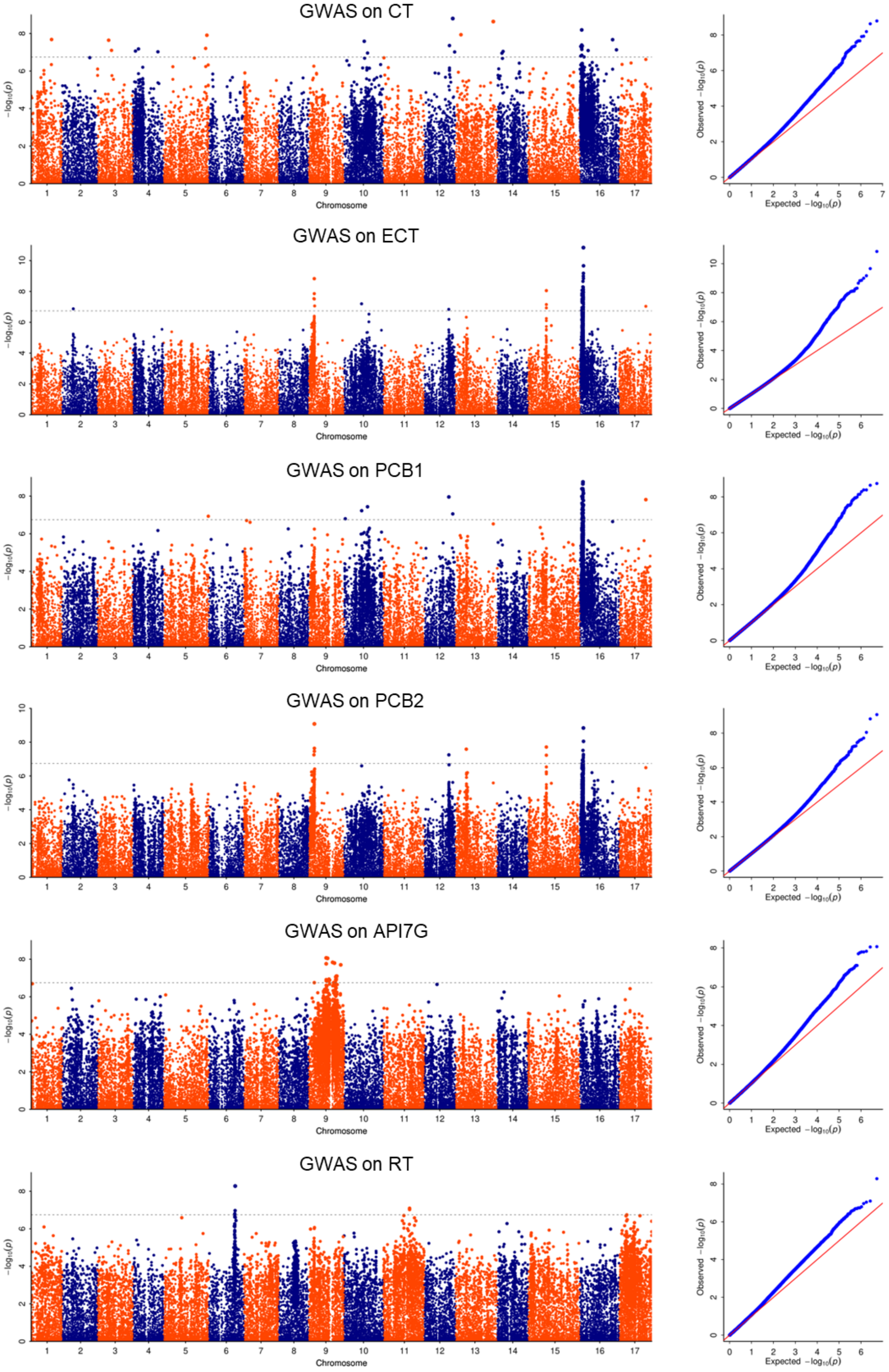
GWAS on CT
GWAS on ECT
GWAS on PCB1
GWAS on PCB2
GWAS on API7G
GWAS on RT
Chromosome
−log10(p)
Expected −log10(p)
Observed −log10(p)

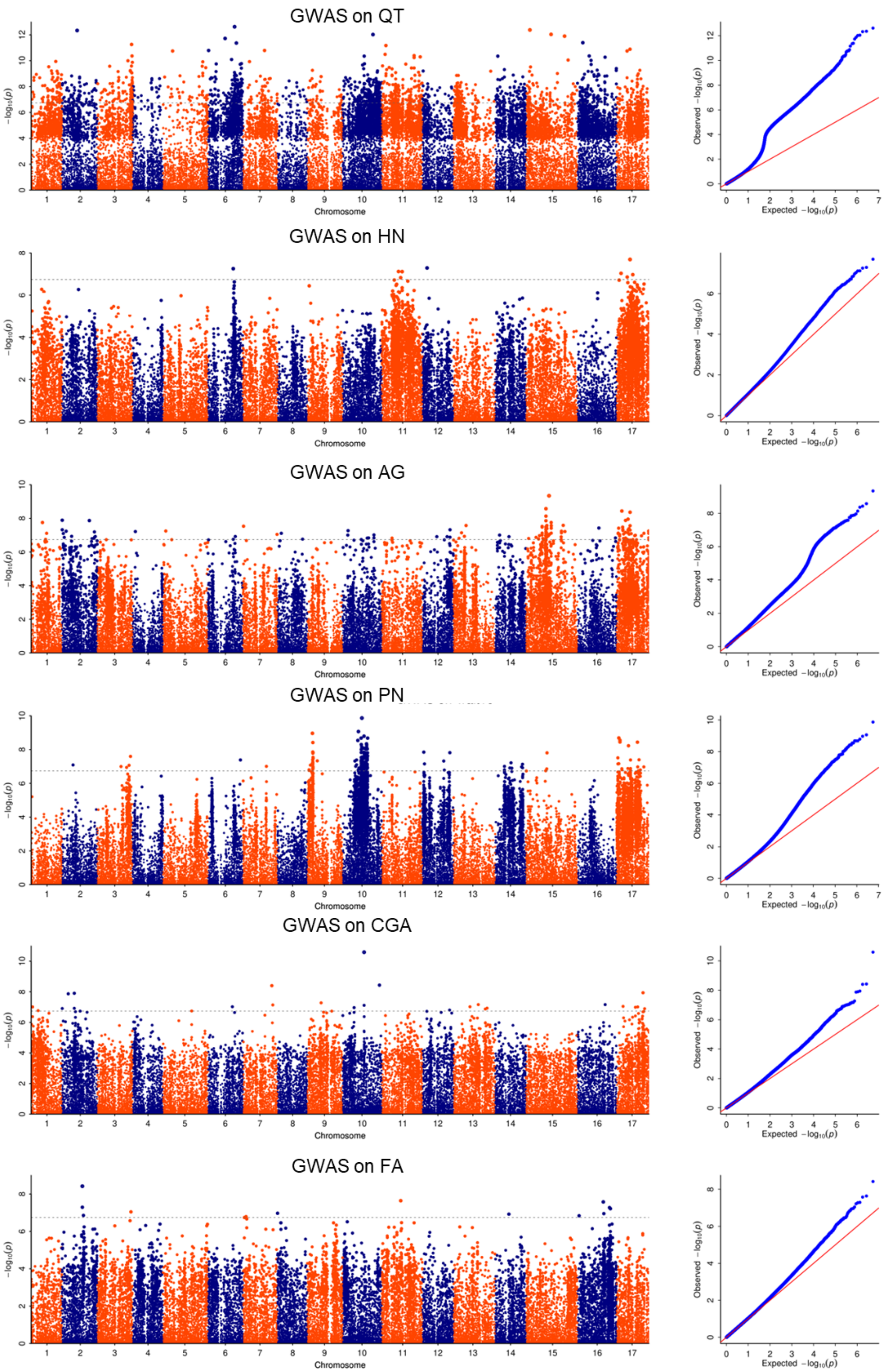
GWAS on QT
GWAS on HN
GWAS on AG
GWAS on PN
GWAS on CGA
GWAS on FA
Chromosome
−log10(p)
Observed −log10(p)
Expected −log10(p)

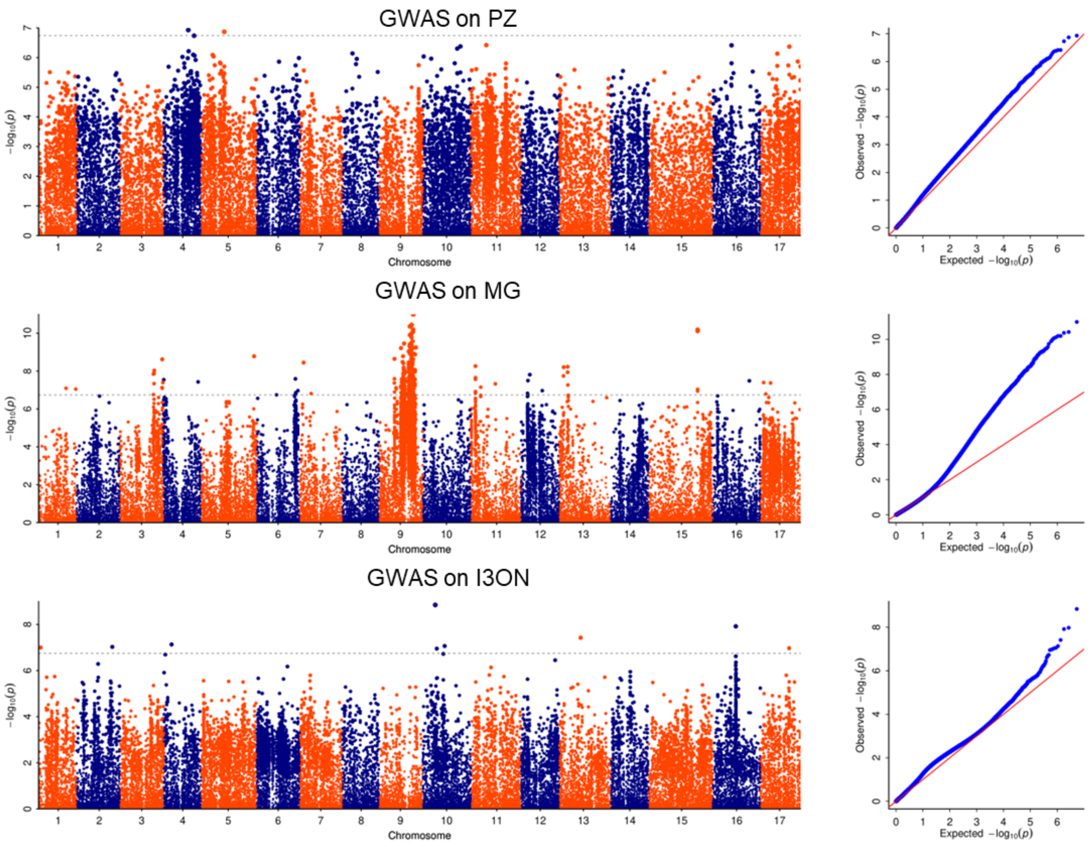

Fig. S5. Manhattan plots and quantile-quantile (Q-Q) plots for 15 individual polyphenol content traits. CT, catechin; ECT, epicatechin; PCB1, procyanidin B1; PCB2, procyanidin B2; API7G, apigenin 7-glucoside; RT, rutin; QT, quercetin; HN, hyperin; AG, astragalin; PN, prunin; CGA, chlorogenic acid; FA, ferulic acid; PZ, phloridzin; MG, methyl gallate; I3ON, isorhamnetin 3-O neohesperidoside.

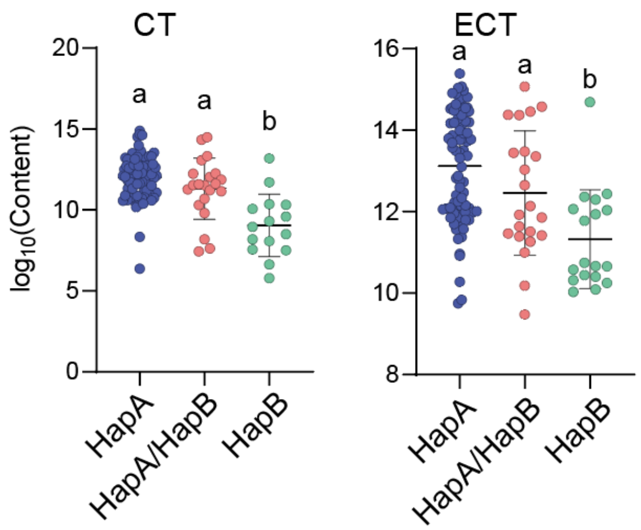

Fig. S6. Boxplots for catechin (CT) and epicatechin (ECT) content followed the haplotypes in Fig. 4J.

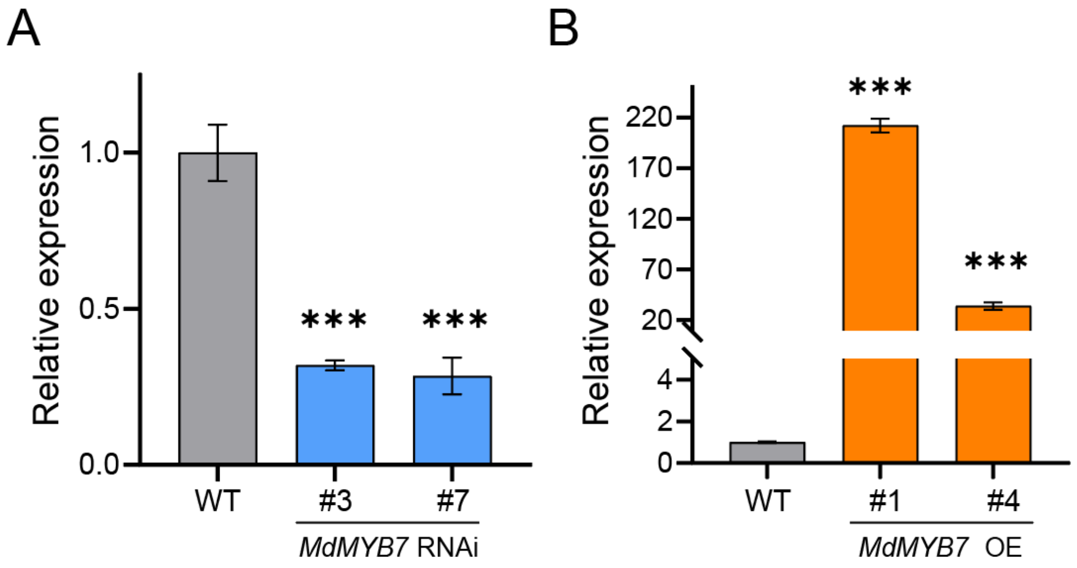

Fig. S7. Relative expression of *MdMYB7* in *MdMYB7* RNAi (A) and *MdMYB7* OE (B) calli by qRT-PCR. Statistical analyses were performed by the student's *t* test and statistically significant differences are indicated by ****P*<0.001.

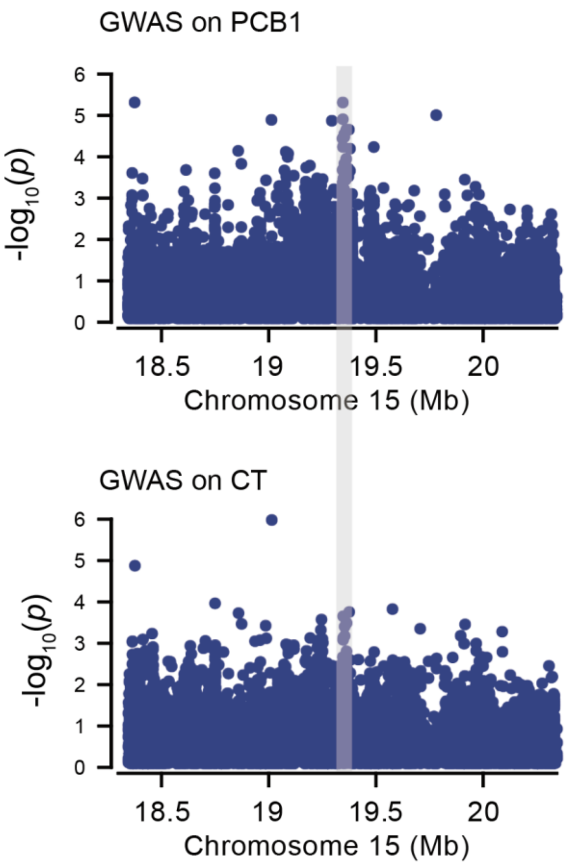

Fig. S8. Local Manhattan plot surrounding the GWAS signal for procyanidin B1 (PCB1, top) and catechin (CT, bottom).

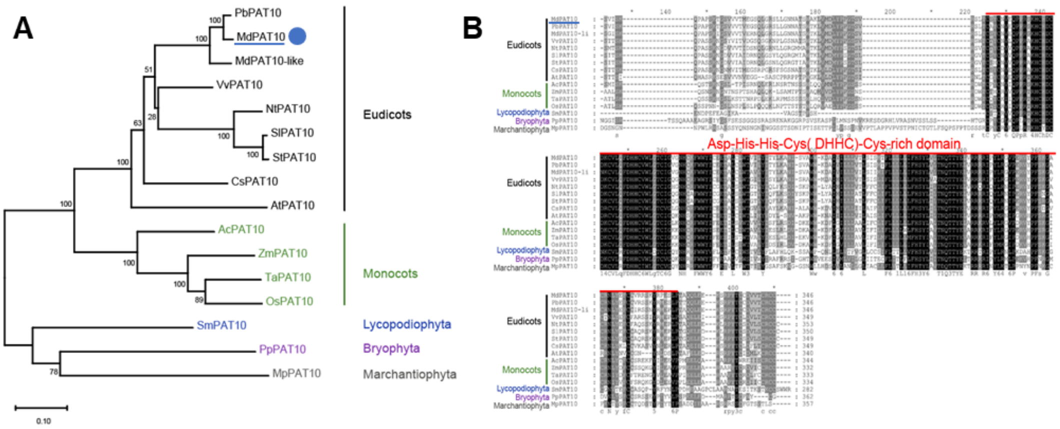

Fig. S9. PAT10 protein sequence analysis in several plant species. (**A**) Phylogenetic tree of *PAT10*. (**B**) Multiple sequence alignment analysis of *PAT10*.

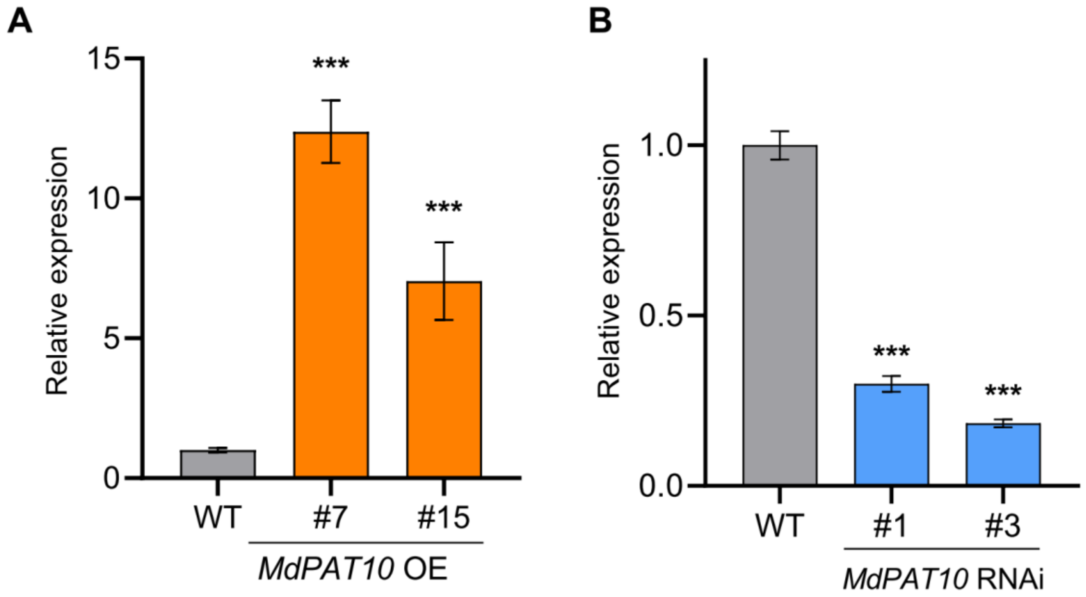

Fig. S10. Relative expression of *MdPAT10* in *MdPAT10* OE (A) and *MdPAT10* RNAi (B) calli by qRT-PCR. Statistical analyses were performed by the student's *t* test and statistically significant differences are indicated by ****P*<0.001.

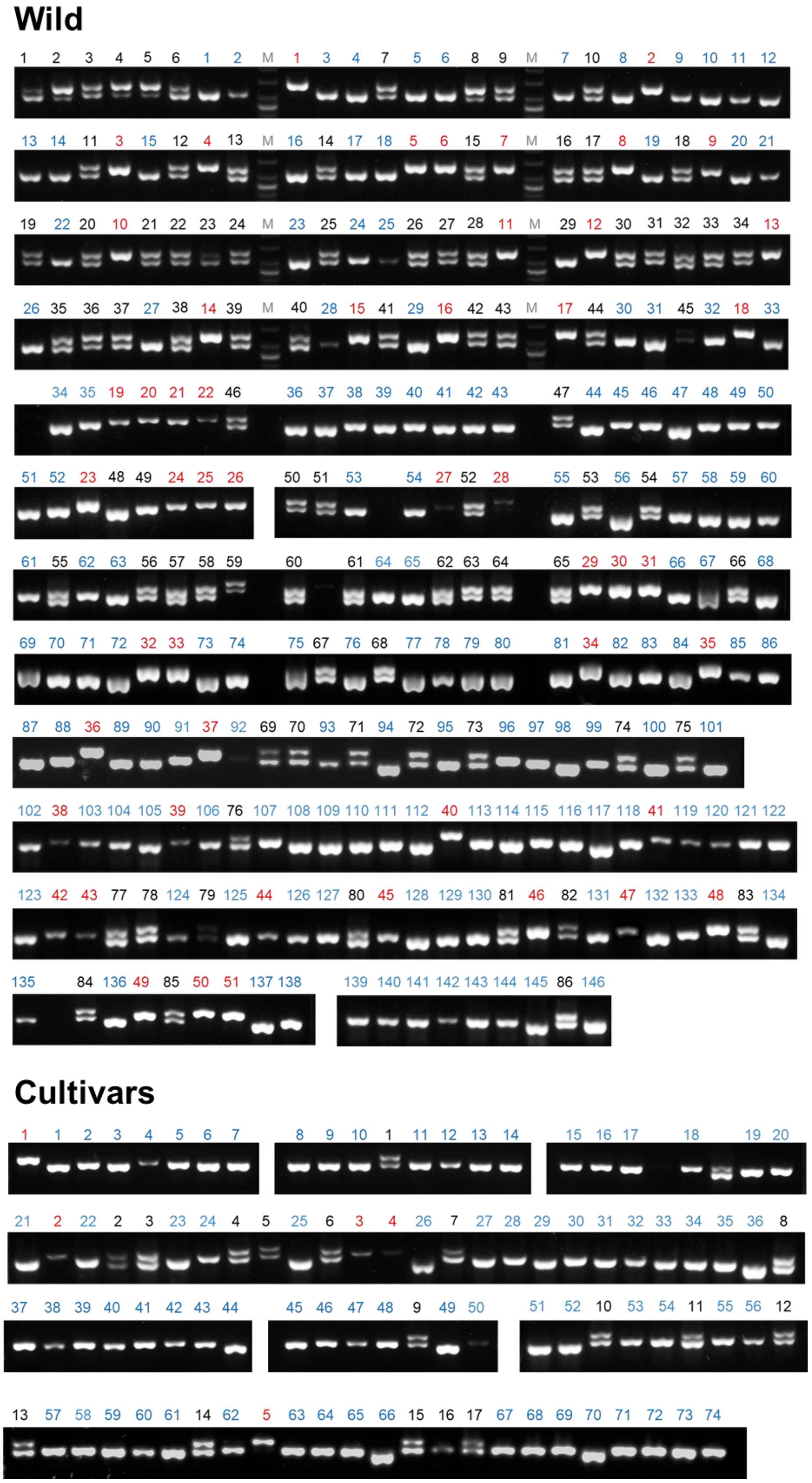

Fig. S11. Detection of the In-868 variation in the *MdPAT10* promoter in wild species and cultivated varieties. Numeric characters, sample

codes; red, homozygous In-868; blue, without homozygous In-868; black, heterozygous In-868; ‘M’, DNA ladder.
